# Suicidal Human Poisoning With Fungicide: A Rare Case From Rural India

**DOI:** 10.7759/cureus.86943

**Published:** 2025-06-28

**Authors:** R Sivasankary, Muthunagai R, Anubhuti Tyagi

**Affiliations:** 1 Forensic Medicine, All India Institute of Medical Sciences, Rishikesh, Rishikesh, IND; 2 Oral and Maxillofacial Surgery, Seema Dental College and Hospital, Rishikesh, IND

**Keywords:** carbendazim, forensic toxicology, fungicide poisoning, mancozeb, pesticides

## Abstract

Fungicide poisoning is a significant concern in agricultural regions, where access to toxic chemicals is common. The aim is to present a case of poisoning from exposure to a highly toxic fungicide, highlight its continued use and availability, and urge public health authorities to act quickly. A 50-year-old male, a milk vendor with a history of diabetic ulcer and chronic low backache, was found unconscious at home with evidence of poisoning. He was declared dead on arrival at the emergency medical department. Reliable information from relatives and the presence of the poison container indicated possible fungicide ingestion. Postmortem examination and subsequent laboratory analyses confirm the poisoning. This case demonstrates the critical importance of thorough forensic investigation, corroborated by laboratory analysis, in determining the cause of death in suspected poisoning cases. It also highlights the need for public awareness regarding the hazards of fungicide exposure and improved safety measures to prevent such incidents.

## Introduction

Self-poisoning with agrochemicals has emerged as a significant and preventable cause of morbidity and mortality in India. Fungicides are widely used in agriculture and animal health to control fungal infections and to prevent their growth [1]. In the majority of reported cases of poisoning, organophosphates are commonly involved; fungicide poisoning remains underrecognized. Compounds such as carbendazim and mancozeb, despite being perceived as safe, have been associated with endocrine disruption, oxidative stress, and organ damage [2]. Their easy availability and limited regulation in rural areas increase the risk of both accidental and intentional exposure. Belpoggi’s study involving clinical observations from hospitals in India documented predominantly mild gastrointestinal symptoms in fungicide poisoning cases, with most patients achieving full recovery, signifying relatively low clinical lethality [3]. However, this should not undermine the potential for severe outcomes in certain contexts. The present case highlights the need for increased awareness and a more robust clinical and forensic approach to fungicide-related toxicity.

## Case presentation

**Figure 1:**
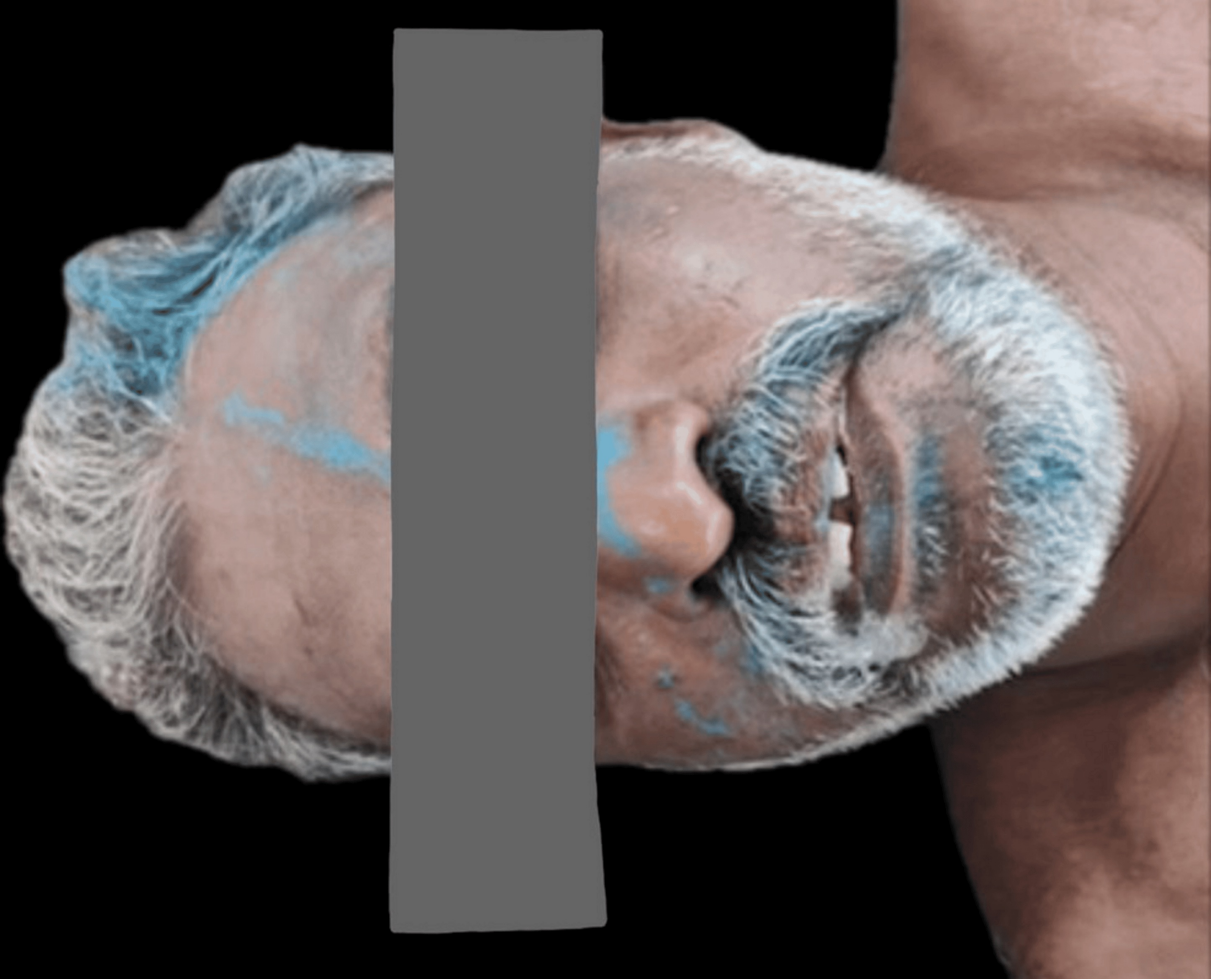
Dried blue stains are visible on the hair, forehead, nose, and around the mouth.

**Figure 2:**
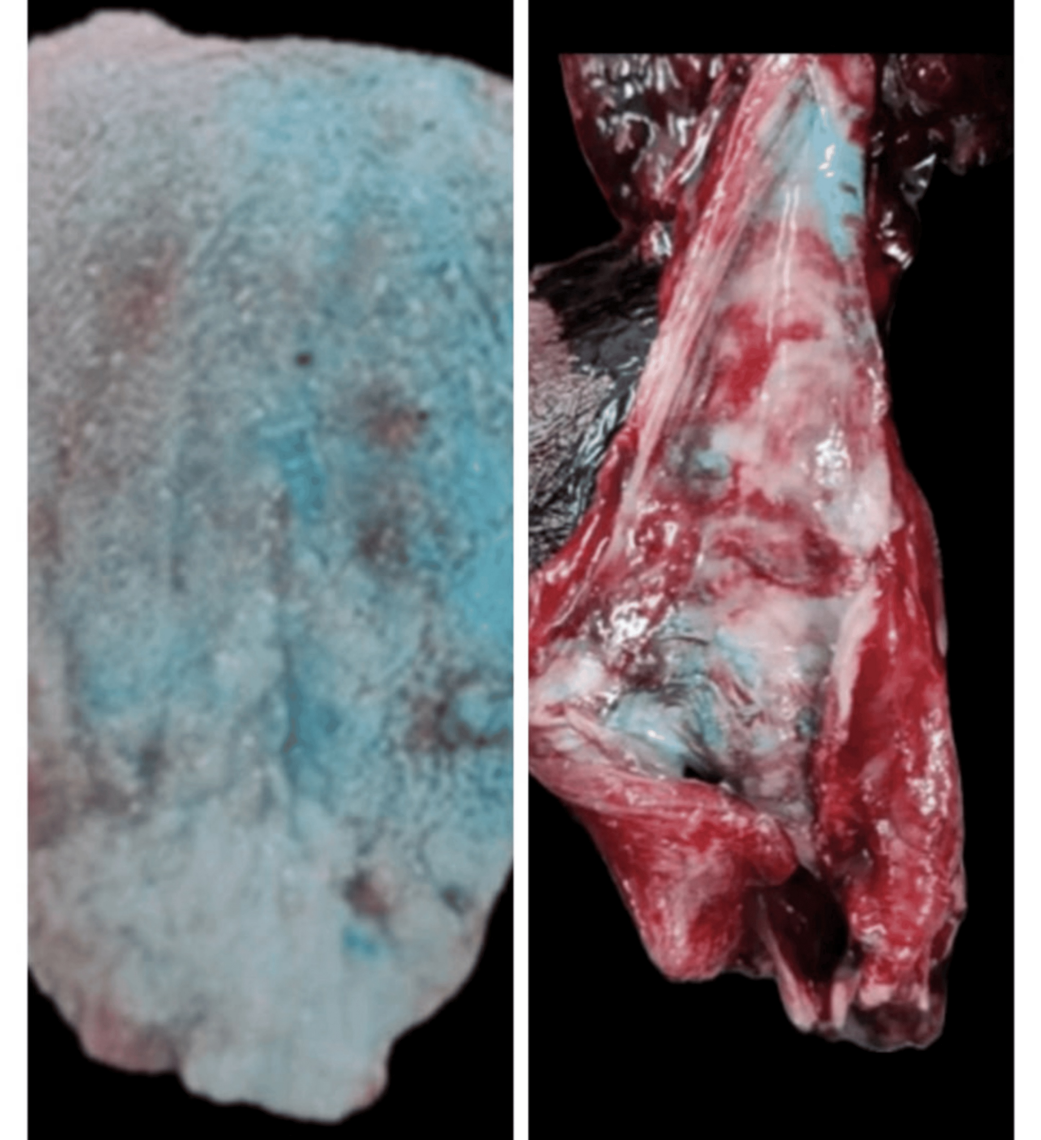
Tongue (left): Exhibits blue staining. Esophagus (right): Presence of blue-colored fluid in areas. The mucosa is congested and edematous.

A 50-year-old gentleman, a milk vendor by occupation, was suffering from a diabetic ulcer and chronic low backache. He was found in an unconscious state with evidence of poisoning at his residence, following which his relatives called the 108 ambulance service. He was brought to the emergency department on 08/05/2023, where he was declared dead on arrival. On 08/05/2023, an autopsy was performed. On examination, rigor mortis was present, postmortem lividity was not fixed, and dried stains of blue colour fluid were present over the face, nose, and areas around the mouth. Eyes are closed, and on opening the eyes, the cornea is hazy, and the tongue shows a blue-colored stain, with a pungent odor. An ulcer is present over the right foot dorsal aspect, measuring 10 cm x 5 cm in midline, 5 cm above the medial malleolus, with a layer of pale yellow color slough and foul-smelling odor, and margins are swollen - grade 2 diabetic ulcer (according to Wagner diabetic foot ulcer classification). On internal examination, blue-colored fluid with a pungent smell is present throughout the oesophagus, stomach, and duodenum. In the oesophagus, the mucosa is edematous and congested. The gastric mucosa is congested and edematous, with multiple petechial hemorrhages present along the lesser curvature, and contains 200 g of blue color undigested food with a pungent odor. Figures 1-2 show the presence of a blue-colored poisonous substance in the gastrointestinal tract. The lungs showed gross congestion, subpleural petechiae, and hemorrhagic pulmonary oedema. The heart was soft and flabby, with petechial hemorrhages in places. The brain was congested and edematous. The meninges were congested. Viscera and blood samples were collected and sent to the forensic science laboratory (FSL). The FSL report confirmed the presence of carbendazim and mancozeb in the content of the stomach and intestines, as well as in the liver and kidneys. A verbal autopsy was conducted with the relatives before autopsy and revealed that the deceased was under stress and experienced loneliness due to his spouse's demise. The FSL report, the history provided by relatives, and the presence of a substance-containing bottle all strongly suggest the ingestion of a fungicide.

## Discussion

Suicide is a major public health issue in India [4]. Statistical data from the National Crime Records Bureau (NCRB) show that there is a consistent surge in suicidal rates from 9.9 per lakh population in 2017 to 12.4 per lakh in 2022, with Sikkim recording the highest at 43.1 per lakh population and Bihar recording the lowest suicidal rate at 0.6 per lakh population [5]. Previously, attempting suicide was considered a criminal offence under section 309 Indian Penal Code (IPC); later, the Mental Healthcare Act of 2017 decriminalized suicide attempts by acknowledging mental distress. Despite this progress, section 309 IPC has not been formally repealed, resulting in legal ambiguity [6-9]. Poisoning is considered one of the top three methods of suicide by the World Health Organization (WHO). In records, most common pesticide-related deaths are due to ingestion of organophosphorus insecticides [10-13]. Pesticides include all compounds that are applied to destroy and regulate pests; they include insecticides (insects), herbicides (weeds), and fungicides (fungi) [14]. Fungicides are less studied in terms of their effect on human health. Generally, fungicides are considered less toxic to mammals due to limited common biological targets; many studies showed that fungicides still cause harm through different mechanisms [15].

The incidence and epidemiology of fungicide poisoning in India are underrepresented in epidemiological records, often subsumed under general pesticide poisoning statistics. Surveillance data from the National Poison Information Centre (NPIC), All India Institute of Medical Sciences (AIIMS), India, indicate that fungicides constitute approximately 5-10% of pesticide-related poison calls, reflective of both accidental and incidental exposures, primarily in agrarian states such as Andhra Pradesh, Maharashtra, Tamil Nadu, and Punjab [16,17]. The incidence is mainly due to easy accessibility and limited regulation enforcement, particularly in rural farming communities.

Fungicides are classified based on the mode of action, general use, and chemical nature, as presented in Table 1 [18].

**Table 1:** Classification of fungicides. MBC: Methylbenzimidazole carbamate; Dithane M 45: M refers to Mancozeb (active ingredient)

Mode of Action	General Use	Chemical Use
Protectants - Sulfur, Captan, Thiram, Zineb, Mancozeb. Therapeutants- Oxathins, Benzimidazoles, Bordeaux mixture, Antibiotics. Systemic - Oxathin, Benzimidazoles, Metalaxyl, Thiophanate, Antibiotics. Non-systemic - Sulphur, Copper Quinone, Hetero nitrogenous group	Seed Protectants - Captan, Thiram, Bavistin, etc. Soil Protectants - Chloroform, Formaldehyde, Vapam, Captan, Thiram, Topsin, etc. Foliage & Blossom protectant - Dithane M-45, Bordeaux Mixture, Bavistin, Blitox, etc. Fruit Protectants: Captan, Difolatan, Dithane M-45, etc. Tree Wound dressing: Bordeaux pastes, Chaubattia paste, etc. Eradicants - Copper, Lime sulphur, etc.	Sulphur fungicide - Powdered Sulphur, Wettable Sulphur, Lime Sulphur, Ferbam, Ziram, Thiram, Zineb, Maneb, Nabam, Vapam, etc. Copper fungicide - Bordeaux mixture, Burgundy mixture, copper oxychloride, etc. Mercural fungicides - Mercuric and Mercurous Chloride, Phenyl Mercury Acetate, Methoxy Ethyl Mercury Chloride, etc. Heterocyclic nitrogenous compounds - Captan, Folpet, etc. Quinone compounds - Chloranil, Dichlone, etc. Oxathiin compounds - Carboxin, Oxycarboxin, etc. Benzimidazoles/Carbendazim group - Benomyl, MBC, etc.

In this case, the FSL report shows that both carbendazim and mancozeb were found in the gastric content, liver, and kidney (blood level concentrations were not mentioned).

Carbendazim, chemically known as methyl benzimidazol-2-ylcarbamate (C₉H₉N₃O₂), is a white crystalline solid with low water solubility. It is stable under standard conditions but degrades in acidic or alkaline environments. As a broad-spectrum systemic fungicide, it is widely used in cereals, fruits, vegetables, ornamental plants, and in seed treatment. Carbendazim works by inhibiting β-tubulin polymerization, disrupting fungal cell mitosis, and eventually causing cell death. In humans, it causes CYP1A2-mediated bioactivation, protein adduct formation, oxidative damage, and endocrine disruption [3,19,20]. Oral LD50 in rats is 6400-15,000 mg/kg. Dermal LD50 in rabbits is more than 2,000 mg/kg, but low acute toxicity in humans [3,21]. Acute toxic effects cause gastrointestinal irritation (nausea, vomiting), CNS depression. Although it has low acute toxicity, prolonged exposure may lead to liver and kidney damage and reproductive effects. It is classified as a possible human carcinogen (group 2B by the International Agency for Research on Cancer (IARC)). Treatment is only supportive care since no antidote is available. A combination with other fungicides and pesticides potentiates toxicity [20].

Mancozeb, a combination of manganese and zinc with ethylene bis (dithiocarbamate) (C₄H₆MnN₂S₄Zn), is a yellowish powder that is not water soluble but disperses easily in water mixtures. It is stable under normal conditions and breaks down under heat and moisture, releasing ethylene thiourea (ETU), a known toxin. The mechanism of action is by disrupting fungal enzyme activity through interaction with sulphhydryl groups, leading to the inhibition of various metabolic processes. Animal LD50 in rats is approximately 4,500-11,000 mg/kg. Dermal LD50 in rabbits is more than 10,000 mg/kg. No direct LD50 was measured in humans due to ethical constraints [18,22]. Acute poisoning manifests with gastrointestinal irritation, central nervous system depression, and allergic reactions. Chronic exposure causes thyroid dysfunction and neurodevelopmental impacts [3,22]. Supportive care is needed as a treatment, as no antidote is available.

Diagnosing remains challenging as conventional immunoassays do not reliably detect these compounds. Hence, advanced techniques such as liquid chromatography tandem mass spectrometry (LC-MS/MS) or gas chromatography-mass spectrometry (GC-MS) for targeted identification and quantification in biological specimens such as blood, urine, and gastric contents are needed [23].

Belpoggi et al. reported a rare lethal case of a 45-year-old male who attempted suicide by ingesting carbendazim with monocrotophos, a highly toxic organophosphates. Death occurred after 48 hours of ingestion. In this report, it was said that carbendazim alone was unlikely to be the sole cause of death, but may have contributed to systemic toxicity [3]. A case of deliberate ingestion of carbendazim (formulation up to 200-250 mL of 50% solution) led to gastrointestinal and CNS symptoms; however, complete recovery was achieved with supportive care [3]. A fatal systemic toxicity was reported in a 39-year-old farm worker in Colombia who died after prolonged inhalation of mancozeb dust without personal protective equipment, which led to respiratory failure and CNS depression. Autopsy showed lung inflammation consistent with fungicide inhalation injury [3]. A study conducted by Mesnage et al. concluded that fungicides were the most toxic from concentrations 300-600 times lower than the agricultural dilutions, followed by herbicides and then insecticides [24].

India regulates fungicides under the Insecticide Act 1968, with oversight from the Central Insecticides Board & Registration Committee (CIBRC). Despite the ban on carbendazim and mancozeb, these fungicides remain registered and legally marketed across India as of 2024. Although these drugs are banned, carbendazim and mancozeb were not mandatorily restricted nationally, showing the agricultural dependence and emerging poisoning cases [16,20].

## Conclusions

Although fungicides are generally considered less lethal, they can still cause significant acute and chronic health effects. Clinicians and forensic experts should thoroughly evaluate suspected cases of fungicide toxicity - regardless of perceived low risk - and report them to the appropriate authorities. Public health agencies must actively promote awareness regarding the safe use, handling, and potential short- and long-term effects of fungicide exposure. Moreover, regulations banning harmful fungicides such as carbendazim and mancozeb must be strictly enforced to prevent misuse and protect public health.
